# Characteristics of macular edema associated with retinal vein occlusion showing poor anatomic response to three loading anti-vascular endothelial growth factor injections: an optical coherence tomography analysis

**DOI:** 10.1186/s12886-024-03298-9

**Published:** 2024-01-22

**Authors:** Mohammadkarim Johari, Alireza Attar, Dorna Eghtedari, Seyed Ahmad Razavizadegan

**Affiliations:** Poostchi Ophthalmology Research Center, Department of Ophthalmology, School of Medicine, Shiraz University of Medical Sciences, Shiraz, Iran

**Keywords:** Retinal vein occlusion, Cystoid macular edema, Anti–vascular endothelial growth factor

## Abstract

**Purpose:**

To analyze the clinical features of refractory cystoid macular edema related to retinal vein occlusion associated with the response to three consecutive loading doses of anti–vascular endothelial growth factor.

**Methods:**

A retrospective chart review was performed on retinal vein occlusion patients treated by three anti–vascular endothelial growth factor injections. They were divided into a group according to resolution of macular edema in optical coherence tomography (Group 1) and with persistent macular edema (Group 2). We analyzed qualitative and quantitative morphologic features of optical coherence tomography.

**Results:**

We enrolled a total of 120 eyes from 120 patients (Group 1: *n* = 54, Group 2: *n* = 66). The baseline choroidal thickness differed significantly between groups 1 and 2 (290.70 ± 19.58 μm and 311.06 ± 17.87 μm *P* < 0.001). The presence of Hyperreflective foci (16.70% vs. 36.40% *P* < 0.001), Disorganization of the retinal inner layers (14.80% vs. 87.90%) and external limiting membrane disruption (16.60% vs. 39.3% *P* < 0.001) differed significantly. Logistic regression analysis showed that the initial central macular thickness (B = 0.012; *P* = 0.006), baseline choroidal thickness (B = 0.232; *P* = 0.016) and presence of hyperreflective foci (B = 1.050; *P* = 0.019), disorganization of the retinal inner layers (B = 1.132; *P* = 0.001) and external limiting membrane disruption (B = 1.575; *P* = 0.012) significantly affected the anti–vascular endothelial growth factor treatment response.

**Conclusion:**

A thicker sub-fovea choroid and the presence of hyperreflective foci, disruption of the external limiting membrane and disorganization of the retinal inner layers associated with a poorer response to three loading anti–vascular endothelial growth factor injections in macular edema associated retinal vein occlusion.

## Introduction

Retinal vein occlusion (RVO) is the second most common reason behind retinal vascular disease. Both branch RVO (BRVO) and central RVO (CRVO) are correlated to vision loss and reduction in vision-related quality of life [1]. It causes an increase in pressure within capillaries and veins, leading to the collapse of vessel barriers and the release of blood or plasma components into surrounding tissue, resulting in edema. Macular edema (ME) is a significant complication of RVO that causes severe visual impairment [1]. The emergence of therapies targeting vascular endothelial growth factor (VEGF), known as anti-VEGF therapies, has significantly enhanced the functional and structural results for individuals affected by retinal vein occlusion. Despite this improvement, the response to treatment can vary widely, leading to several studies exploring biomarkers that can predict treatment response [2–4]. After receiving multiple intravitreal injections of anti-VEGF treatment, some patients experience no improvement in macular edema, while others have resolved completely. Spectral domain optical coherence tomography (OCT) is a common diagnostic imaging method used to examine macular structures. OCT can generate cross-sectional images of the retina, which can provide both qualitative and quantitative information about the retina and choroid. For many retinal specialists, the OCT images are essential in making treatment decisions as they can reveal various morphological features such as intraretinal fluid (IRF), subretinal fluid (SRF), hyperreflective foci (HF) and disorganization of the retinal inner layers (DRIL).

As a result, the objective of this study was to examine the clinical characteristics of patients with macular edema secondary to retinal vein occlusion (RVO) who had an inadequate response to treatment and still had remaining fluid visible on OCT images after three consecutive loading doses of anti-VEGF. The goal was to compare the qualitative and quantitative morphological features of OCT at baseline between groups with and without residual fluid. Identifying these factors could help clinicians to better manage patient expectations and determine the most appropriate treatment approach for these individuals.

## Method

### Patients

The research conducted in this study followed the principles outlined in the Declaration of Helsinki and was authorized by the institutional review board of Shiraz University of medical science. As the study was conducted retrospectively, the necessity for informed consent was waived. We conducted a thorough examination of the medical records of patients who were diagnosed with cystoid macular edema caused by retinal vein occlusion and had not previously undergone treatment. The study was carried out at our clinic between January 2018 and January 2021. We included patients who received three monthly intravitreal anti-VEGF treatments (bevacizumab 1.25 mg/0.05 cc StivantR, CinnaGen Co., Iran) and analyzed the OCT results one month after the final injection. Patients were divided into two groups based on the OCT images: Group 1, eyes with resolution of ME and Group 2 if ME was present. Resolution of ME was defined as CST less than 250 μm, no subretinal or intraretinal fluid, and no cystoid spaces within the ETDRS grid based on OCT imaging.

## Data collection

We gathered patient demographic information, past medical history, axial length and results from ophthalmic examinations, and OCT images. We also recorded the type of retinal vein occlusion, which included branch RVO (BRVO) and central RVO (CRVO), we considered hemi-RVO (HRVO) as BRVO in analyses. OCT measurements were recorded at baseline and one month after the third injection.

We excluded patients who had previously received treatment for their macular edema, including laser photocoagulation, anti-VEGF injection, subtenon or intravitreal steroid injection, or had undergone pars plana vitrectomy. Additionally, we excluded patients who had other retinal diseases that could potentially cause macular edema, such as diabetic retinopathy, age-related macular degeneration, and macular pucker.

### Optical coherence tomography

We used the Spectralis OCT instrument (Spectralis OCT; Heidelberg Engineering, Heidelberg, Germany) to perform spectral domain OCT and enhanced depth imaging optical coherence tomography (EDI-OCT). The light source was centered at 870 nm, and the axial and transverse resolutions for tissue imaging were 3.9 and 6 mm, respectively. We evaluated various measurements, including central macular thickness (CMT), sub-fovea choroidal thickness (SFCT), hyperreflective foci (HF), and the disruption of external limiting membrane (ELM) and disorganization of the retinal inner layers (DRIL).

**Fig. 1:**
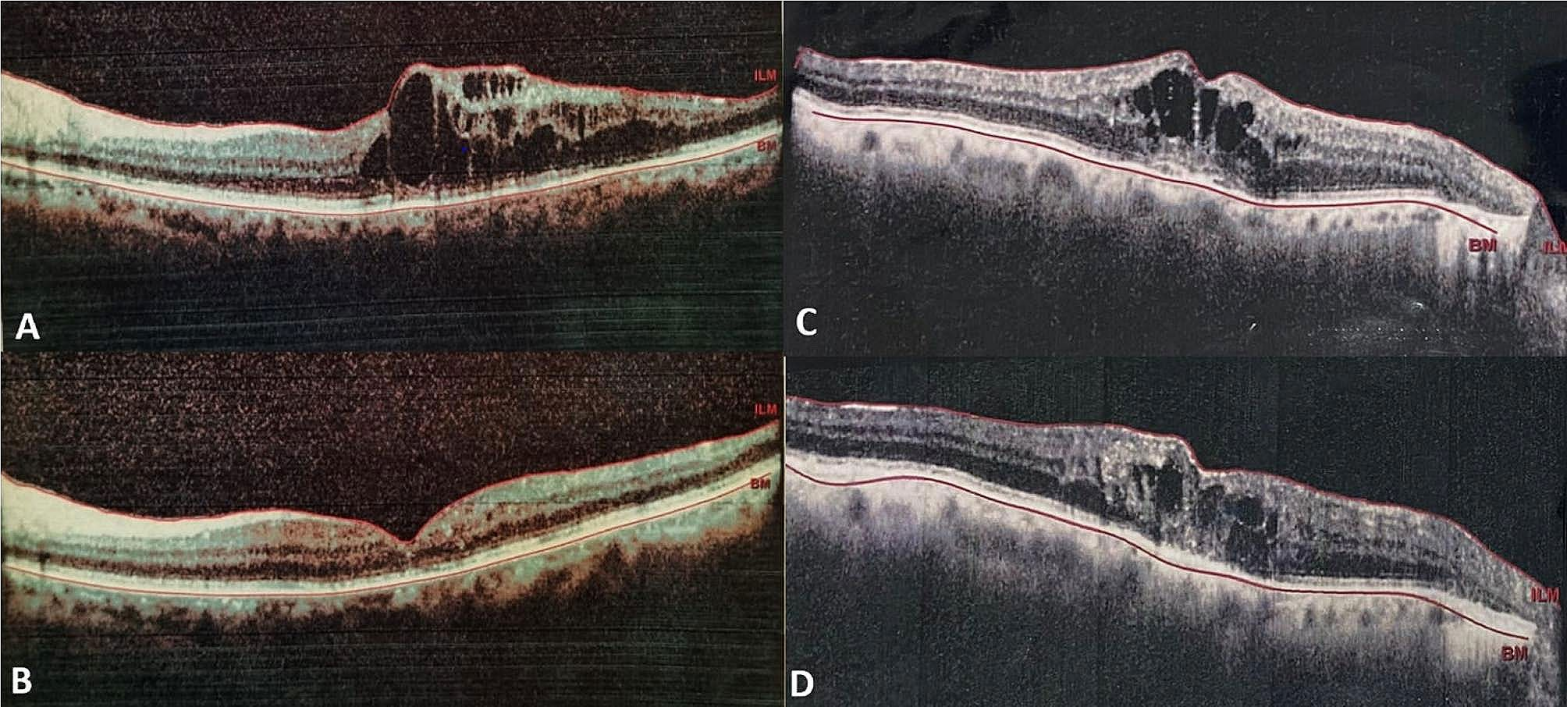
Optical cohierance tomography of patient in group 1, which shows resolution of macular edema after three loading anti-VEGF injection, (**A**-**B**), and in group 2 which shows persistence of macular edema after three loading anti-VEGF injection (**C**-**D**).

Central macular thickness was calculated as the average macular thickness in the central 1-mm Early Treatment Diabetic Retinopathy Study grid, using the instrument’s software. Sub-fovea choroidal thickness was measured in RVO eyes and fellow eyes as an average value by determining the perpendicular distance from the outer layer of the retinal pigment epithelium to the inner surface of the sclera in the subfoveal area, utilizing software calipers. Hyperreflective foci were identified as tiny, well-circumscribed, dot-shaped lesions with reflectivity equal to or greater than the RPE band. We categorized the location of the foci in the retina as inner retina (from the outer nuclear layer to the internal limiting membrane) or outer retina (from above the retinal pigment epithelium to the external limiting membrane). External limiting membrane (ELM) is a distinctive feature of photoreceptor function and is regarded as the zonula that adheres between photoreceptors and Müller cells. Disorganization of the retinal inner layers (DRIL) was defined as the horizontal extent of the disarray between the ganglion cell–inner plexiform layer complex, inner nuclear layer, and outer plexiform layer (Fig. 1).

### Statistical analyses

The statistical analysis was conducted using IBM SPSS Statistics Version 23.0 (IBM Corp., Armonk, NY, USA). The results are expressed as mean ± standard deviation (SD). To compare categorical variables between the two groups, the Pearson chi-square test was employed. For continuous variables, the Student t-test was used for analysis. To identify factors that could potentially affect the response to anti-VEGF treatment, both univariate and multivariate logistic regression analyses were performed. A P-value less than 0.05 was considered statistically significant.

## Results

A total of 120 eyes from 120 patients with RVO related macular edema and receiving anti-VEGF treatment were included in this study. 70 eyes were diagnosed as CRVO and 50 eyes as BRVO or hemi-RVO. We reviewed 54 (45%) eyes of the group resolved ME (Group 1) and 66 (55%) eyes of the group with persistent ME (Group 2). The baseline characteristics of the patients are listed in Table 1. The mean ages were 63.00 ± 11.32 and 64.73 ± 11.85 years, respectively. The mean axial length was 23.73 ± 6.81 mm and 23.34 ± 6.32 mm in Group 1 and 2, respectively. The mean spherical equivalent was 0.13 ± 1.50 and 0.25 ± 1.31 diopters, respectively, these differences were not significant (*P* > 0.05).

**Table 1:** Baseline characteristics of patients

Variables	Group 1 (n = 54)	Group 2 (n = 66)	P value
Age (years, mean ± SD)	63.00 ± 11.32	64.73 ± 11.85	0.945**
Sex (M/F)	28/26	40/26	0.336*
IOP (mmHg, mean ± SD)	15.85 ± 2.31	17.67 ± 2.63	0.001**
S.E (D, mean ± SD)	0.13 ± 1.50	0.25 ± 1.31	0.462**
Axial length (mm, mean ± SD)	23.73 ± 6.81	23.34 ± 6.32	0.902**

P-value * is calculated by Chi-Square Test, P-value ** is calculated by two independent sample T-Test

D, diopters; IOP, intraocular pressure; S.E, spherical equivalent

The initial and final CMT values were 408.22 ± 127.93 μm and 277.15 ± 45.36 μm in group 1, and 705.39 ± 244.69 μm and 499.64 ± 148.88 μm in group 2. The baseline SFCT values in RVO eyes were 290.70 ± 19.58 μm and 311.06 ± 17.87 μm, respectively; this difference was significant (*P* < 0.001). The baseline SFCT values in fellow eyes were 272.35 ± 13.52 μm in group 1, and 280.13 ± 23.12 μm in group 2. the baseline SFCT in RVO eyes was significantly thicker than that in contralateral eyes in both groups (*P* < 0.001) although this difference was not significant between groups (*P* > 0.05). Hyperreflective foci were present in 16.70% and 36.40% of the eyes in Groups 1 and 2, respectively. In Group 1, the proportions of cases with HF involving inner retina or outer retina were 10.50% and 6.30%, respectively. In Group 2, the proportions of cases with involvement of inner retina or outer retina were 16.80%, 19.60%, respectively, this difference was significant (*P* < 0.001). ELM disruption was present in 16.60% and 39.3% of the group 1 and 2 patients, respectively, and the presence of DRIL was detected in 14.80% and 87.90% of the group 1 and 2 patients, with significant P values *P* < 0.001 respectively. These values, measured manually by the two graders, exhibited excellent interobserver reproducibility (intraclass correlation coefficient. 0.95, coefficient of variation, 5%) (Table 2).

**Table 2:** Optical coherence tomography findings of patients

Variables	Group 1 (n = 54)	Group 2 (n = 66)	P value
CMT baseline (mean ± SD)	408.22 ± 127.93	705.39 ± 244.69	< 0.001**
CMT final (mean ± SD)	277.15 ± 45.36	499.64 ± 148.88	< 0.001**
SFCT baseline (mean ± SD)	290.70 ± 19.58	311.06 ± 17.87	< 0.001**
SFCT baseline FE (mean ± SD)	272.35 ± 13.52	280.13 ± 23.12	0.211**
SFCT final (mean ± SD)	283.81 ± 19.27	303.03 ± 17.75	< 0.001**
HF number (%)	9(16.7)	24(36.4)	0.016*
DRIL (number)	8(14.8)	58(87.9)	< 0.001*
ELM disruption (number)	9(16.6)	26(39.30)	< 0.001*
Different CMT (mean ± SD)	131.07 ± 124.28	205.76 ± 137.96	0.003**
Different SFCT (mean ± SD)	6.89 ± 2.93	8.03 ± 4.07	0.087**

P-value * is calculated by Chi-Square Test, P-value ** is calculated by two independent sample T-Test

CMT, central macular thickness; SFCT, sub-fovea choroidal thickness; HF, hyperreflective foci; DRIL, disorganization of the retinal inner layers; ELM, external limiting membrane; FE, fellow eye

After three-loading doses of anti-VEGF, mean CMT and mean SFCT decreased from 571.67 ± 249.07 μm to 399.52 ± 159.305 μm (*P* < 0.001) and 301.90 ± 21.18 μm to 294.38 ± 20.73 μm (*P* < 0.001) respectively, Although decreased in CMT from baseline to last follow-up in groups 2 was significantly higher than group 1 (131.07 ± 124.28 μm, 205.76 ± 137.96 μm *P* = 0.003 group 1,group2 respectively) this value for decreased in CSFT from baseline to last follow-up between groups was not significant (6.89 ± 2.93 μm, 8.03 ± 4.07 μm, *P* = 0.087 group 1, group 2 respectively).

Logistic univariable analysis showed that the baseline CMT value (B = 0.012; *P* = 0.006), baseline SFCT value (B = 0.232; *P* = 0.016) and presence of HR (B = 1.050; *P* = 0.019), DRIL (B = 1.132; *P* = 0.001) and ELM disruption (B = 1.575; *P* = 0.012) were significantly associated with a poor response to three loading anti-VEGF injections in RVO patients. In multivariable analysis, baseline CMT value (B = 0.012; *P* = 0.004), baseline SFCT value (B = 0.112; *P* = 0.003) and the presence of HR (B = 1.819; *P* = 0.005), DRIL (B = 1.342; *P* = 0.012) and ELM disruption (B = 1.431; *P* = 0.002) were significantly associated with residual cystoid macular fluid after anti-VEGF treatment of RVO.

The subgroup analysis revealed that out of the eyes diagnosed with CRVO, 29 (41.40%) were in the group without residual fluid (Group 1), while in the eyes diagnosed with BRVO, 25 (50%) were in the same group (*P* = 0.35). The presence of HF was found in 28.57% of eyes with CRVO and 26% of eyes with BRVO (*P* = 0.574). Regarding the disruption ELM, it was present in 23.65% of eyes with CRVO and 20.23% of eyes with BRVO (*P* = 0.318). The presence of DRIL was detected in 58.57% of CRVO patients and 50% of BRVO patients (*P* = 0.355).

## Discussion

The rationale behind anti-VEGF therapy for ME following RVO is based on the observation of increased intraocular VEGF levels in RVO patients compared to a control group [5, 6]. As a result, anti-VEGF agents have become the standard treatment for ME secondary to RVO in numerous studies [7–10]. Clinical trials conducted for regulatory approval of anti-VEGF as a primary treatment for RVO involved monthly administration for 6 months (loading phase), followed by as-needed administration throughout the first year [7–10]. However, in real-world clinical practice, evidence suggests that anti-VEGF injections are administered less frequently than in the large registration studies [11, 12]. One study, which analyzed health insurance claims from a database covering 64 million individuals in the USA, found that patients who began treatment with bevacizumab for RVO received a mean annual number of injections ranging from only 3.3 to 3.5 [12].

In the present study, we aimed to assess the anatomical effects of three loading doses of anti-VEGF injections in ME related to RVO and also evaluated OCT characteristics that may be associated with a poor response to this treatment.In our study, we observed that 45% of patients with RVO who were treated with bevacizumab achieved a dry macula after three loading injections. It is noteworthy that in all regulatory trials, the maximum decrease in CMT typically occurs after the first anti-VEGF injection. For comparison, in the BRAVO study, around 84.7% of patients achieved a normal CMT of ≤ 250 μm at the 6-month mark [8]. Similarly, in the CRUISE study, approximately 76.9% of patients achieved a CMT of ≤ 250 μm at 6 months [7]. These findings indicate the efficacy of anti-VEGF treatment in reducing CMT in patients with RVO. In our study, we identified that cases with poor responses to the three-loading anti-VEGF treatment had thicker baseline CMT and subfoveal choroidal thickness (SFCT) values. Additionally, we observed the presence of hyperreflective foci (HF), disorganization of the retinal inner layers (DRIL), and disruption of the external limiting membrane (ELM) from OCT images in these patients.

Previous studies have highlighted the importance of SFCT, HF and DRIL as prognostic factors for visual improvement in RVOs treated with anti-VEGF therapy. However, there is currently a lack of research investigating these findings specifically in anatomically responsive cases. Rayess et al. [13] conducted a study and found that a higher baseline SFCT was a positive predictor of favorable visual outcomes in patients with BRVO in their univariate analysis, although it did not hold as a significant predictor in their multivariate analysis. On the other hand, Yu et al. [14] reported that SFCT did not have a significant impact on visual acuity (VA) in patients with RVO. In contrast to these findings, our study revealed that a higher baseline SFCT was associated with a worse treatment response. It is possible that the increase in choroidal thickness, primarily due to stromal edema, exerts additional pressure on the retinal pigmented epithelium (RPE), leading to more ischemia and higher VEGF levels in the retina. Regarding HF, while the exact cause remains unknown, multiple studies have identified HF as a biomarker for poor visual outcomes [15, 16]. In our study, we found that the presence of baseline HF was associated with the need for more anti-VEGF injections. Recent research has suggested that HF may be caused by extravasated lipoproteins, lipid-laden macrophages and microglia in an inflammatory environment, reflecting the breakdown of the blood-retina barrier in RVO, ultimately leading to ME [17]. Furthermore, studies conducted by Babiuch et al. [18] and Chan et al. [19] demonstrated that baseline DRIL correlated with baseline and final VA, in CRVO and hemi-RVO cases, the absence of DRIL at baseline was associated with larger VA improvements at 6 months. However, in our study, we found that the presence of DRIL was a predictor of increased anti-VEGF resistance in RVO treatment.

ELM is considered to be a significant indicator of photoreceptor function, and its condition directly correlates with the capacity for visual function and photoreceptor recovery. Tang et al. [17] conducted a study and discovered that the baseline extent of ELM disruption strongly correlated with VA both at baseline and at the 3-month following anti-VEGF therapy in patients with RVO. Similarly, several other studies have also reported that having an intact ELM at the beginning of treatment predicts better visual outcomes after anti-VEGF medication in RVO patients [15, 20]. In line with these findings, our study revealed that the initial presence of ELM disruption was associated with the CMT after three loading doses of anti-VEGF injections.

The large clinical trials, such as the post hoc analyses of standard care vs. corticosteroid for RVO 1 (SCORE1) and the study of comparative treatments for RVO 2 (SCORE2) studies, have found no significant correlation between initial central macular thickness (CMT) and visual outcomes in RVO eyes treated with grid laser photocoagulation, anti-VEGF therapy, or intravitreal triamcinolone [3, 21]. However, our research yielded different results, as we observed that persistent macular edema (ME) after three loading doses of anti-VEGF injections was more common in the group with higher initial CMT. This finding suggests that the initial CMT could have an impact on the response to treatment. One possible explanation for this association is the effect of mechanical stress on retinal cells, which may influence the duration required for the macular structure to return to normal after injections.

Multiple studies have reported an association between gender and age with final vision outcomes in patients with BRVO. In The HORIZON extension study, it was observed that male patients had a higher likelihood of achieving better vision compared to female patients with BRVO [2]. One possible explanation for this difference is the higher hematocrit range in men, which leads to a stronger oxygen-carrying capacity. This, in turn, helps prevent ischemia and the subsequent elevated levels of VEGF and its related issues. In our study, despite having a larger number of male participants, we did not find a significant difference in anatomical response to three loading injections between genders. Also It is worth noting that the vitreous body and vitreoretinal interface undergo irreversible changes with age, which may impede the diffusion of anti-VEGF agents into the retinal layers. Numerous studies have shown that younger age is associated with better final visual acuity [22, 23]. In our research, we also observed that patients who anatomically responded to three loading doses of anti-VEGF were generally younger than those in the other group. However, this age difference was not statistically significant.

Our study has several limitations, primarily due to its retrospective nature. Firstly, our investigation focused on various characteristics of RVO with only short-term follow-up. Additionally, we solely evaluated anatomical responses based on OCT changes after treatment. To provide more comprehensive insights, future studies should include long-term outcomes, such as best-corrected visual acuity, OCT, and OCTA features. Nonetheless, our study holds significance as we were able to confirm the response during the loading phase, which aids in determining the most appropriate treatment strategy. Secondly, the relatively small sample size in both groups, resulting from strict inclusion criteria, may introduce some bias. To validate our findings and hypotheses more effectively, future studies with a larger number of cases are warranted. Thirdly, it’s essential to consider that real-world settings now offer various anti-VEGF drugs, such as aflibercept or faricimab, for treating ME in RVO patients. These drugs may have different effects and potentially yield diverse results in such cases. However, despite the limitations, our study holds value as it analyzed the characteristics of RVO patients who displayed a poor response to three loading doses of anti-VEGF. This information can be important in understanding treatment outcomes and guiding further research in this field.

## Conclusion

Optical Coherence Tomography proves to be a valuable technique for visualizing the morphological features of the macular edema linked to retinal vascular occlusion. This noninvasive method also aids in predicting the response to anti-VEGF treatment in affected patients. The presence of hyperreflective foci (HF), disruption of the external limiting membrane (ELM), and disorganization of the retinal inner layers (DRIL) were found to be associated with a poor treatment response, as evidenced by the persistence of residual fluid in OCT images following three loading doses of anti-VEGF. Additionally, a thicker sub-foveal choroid showed a significant association with an unfavorable short-term response.

## Data Availability

The datasets generated and/or analyzed during the current study are not publicly available due to the confidentiality of patients’ information but are available from the corresponding author on reasonable request.
